# Insights Into the Neutrophil-Lymphocyte Ratio in Primary Varicose Vein Disease Screening and Prognosis

**DOI:** 10.7759/cureus.71776

**Published:** 2024-10-18

**Authors:** Sanath Patil, Sandhya Nallamotu, Badareesh L, Joanna Sanderwale

**Affiliations:** 1 General Surgery, Kasturba Medical College, Manipal, Manipal, IND; 2 General Surgery, Murrieta Valley Surgery Associates, Temecula, USA; 3 General Surgery, Kasturba Medical College and Kasturba Hospital, Manipal, IND

**Keywords:** chronic venous insufficiency, general surgery, neutrophil-to-lymphocyte ratio (nlr), screening, varicose veins, vascular surgery

## Abstract

Introduction

Varicose veins are a common cause of deterioration in quality of life. Chronic venous disease (CVD) is highly prevalent yet underdiagnosed. This discrepancy in care may change with better insights into the pathophysiological development of CVD.

Objective

In this retrospective study, we aimed to assess the ability of the neutrophil-lymphocyte ratio (NLR), a single inflammatory marker, in screening for primary varicose veins.

Methods

A total of 130 patients aged 21-70 years diagnosed with primary varicose veins from January 1, 2016, to January 30, 2023, were retrospectively studied at the Department of General Surgery, Kasturba Medical College, Manipal. Patients were divided into two groups based on their degree of primary varicose veins using the Clinical-Etiology-Anatomy-Pathophysiology (CEAP) classification. Group 1 included patients with varicose veins with CEAP stage ≤ C3 and group 2 included patients with varicose veins with CEAP stage > C3. Complete blood counts taken at diagnosis were used for NLR calculation.

Results

Absolute neutrophil counts (CI 95%), absolute lymphocyte counts (CI 99%), and NLRs were all statistically significant inflammatory markers in both groups. NLR was found to have a strong statistically significant association with the diagnosis of primary varicose veins (p-value<0.001). NLRs were lower in group 1 than in group 2.

Conclusion

This study conclusively finds that NLR may effectively be considered to track the incidence of primary varicose veins in patients after further studies.

## Introduction

Lower extremity varicose veins affect around 26% of adults and are a common cause of deterioration in quality of life. Chronic venous disease is highly prevalent but underdiagnosed, or diagnosed but not taken seriously till it has a disabling impact on the patient, leaving many patients untreated for many years, some their whole lives [1]. Many patients go untreated for years, and some their whole lives. However, the pathophysiology of CVD is not yet well understood and is an area of active ongoing research. The widely accepted theory on the development of primary varicose veins is colloquially referred to as the leukocyte “trapping” model. Inflammatory mediators are believed to modulate this response [2,3]. In this study, researchers attempted to understand whether reviewing the neutrophil-lymphocyte ratio (NLR) is statistically significant enough to be used in the screening of primary varicose vein disease. A better understanding and being able to quantify the role of inflammation by analyzing the NLR in serum may also give an insight into disease progression in patients with primary varicose veins. With this knowledge, it may be possible to prevent and better treat the root cause of this debilitating disease in the future.

## Materials and methods

This retrospective study was conducted by the Department of General Surgery, Kasturba Medical College, Manipal. A total of 130 patients, aged 21-70 years, of whom 18 were female and 112 were male, diagnosed with primary varicose veins between January 1, 2016, and January 30, 2023, were retrospectively studied. Ethical approval (reference identification: IEC2-649-2023) for the study was granted by the Institutional Ethics Committee of the university. All male and female patients definitively diagnosed with primary lower limb varicose veins from 2016 to the end of 2023 were included in this study. Patients less than 18 years of age or with a limited diagnosis of varicose veins with possible secondary causes were excluded. The sample size was limited by the total number of patients seen by Kasturba Hospital’s Department of General Surgery during the aforementioned time period. Eligible patients’ data were obtained from hospital records retrospectively, and thus patients’ informed written consent to release data publicly was not attainable. To the researchers’ knowledge, all patients included in this study suffered from primary and not secondary varicose veins. The patients were divided into two groups based on their degree of primary varicose veins using the Clinical-Etiology-Anatomy-Pathophysiology (CEAP) classification system for varicose veins. Group 1 included patients with varicose veins with CEAP stage ≤ C3, and group 2 included patients with varicose veins with CEAP stage > C3. Blood samples were taken from the upper extremity with the patient in supine position. Complete blood counts were screened at diagnosis, pre-operatively, with an automated analyzer. Differential leucocyte counts were calculated using microscopy, from which researchers calculated the NLRs. The statistical analysis of these NLR data was done using SPSS for Windows Version 21.0 (IBM Corp., Armonk, NY) and Microsoft Excel (Microsoft Corp., Redmond, WA). Categorical variables were presented in the form of a frequency table. Continuous variables were presented as mean ± SD or median (min, max). Categorical variables were analyzed using the chi-square test. The Shapiro-Wilk test was used to check normality. Numerical data were analyzed using the Student t-test, and nonparametric data were analyzed using the Mann-Whitney U test.

## Results

In this study, we observed that edema, pigmentation, inflammation, induration, itching, fatigue, cramps, ulcers, and bleeding have a significant association with varicose veins in both groups. However, no association was observed with pain in both groups (Table 1).

**Table 1:** Distribution of subjects based on symptoms at presentation in groups 1 and 2 ^C^Chi-square test. *Statistical significance. From the chi-square test, it can be observed that edema, pigmentation, inflammation, induration, itching, fatigue, cramps, ulcers, and bleeding had significant association with varicose veins in both groups. However, no association was observed with pain in both groups.

Variable	Subcategory	Groups		p-Value
		Group 1	Group 2	
		Number of subjects (%)		
Varicosities	Yes	44 (33.8%)	86 (66.2%)	-
Pain	No	15 (42.9%)	20 (57.1%)	0.188^C^
	Yes	29 (30.5%)	66 (69.5%)	
Edema	No	19 (63.3%)	11 (36.7%)	<0.001^C^*
	Yes	25 (25%)	75 (75%)	
Pigmentation	No	40 (88.9%)	5 (11.1%)	<0.001^C^*
	Yes	4 (4.7%)	81 (95.3%)	
Inflammation	No	44 (40%)	66 (60%)	<0.001^C^*
	Yes	0	20 (100%)	
Induration	No	44 (46.8%)	50 (53.2%)	<0.001^C^*
	Yes	0	36 (100%)	
Itching	No	41 (41.4%)	58 (58.6%)	<0.001^C^*
	Yes	3 (9.7%)	28 (90.3%)	
Fatigue	No	43 (36.8%)	74 (63.2%)	0.036^C^*
	Yes	1 (7.7%)	12 (92.3%)	
Cramps	No	41 (39.8%)	62 (60.2%)	0.005^C^*
	Yes	3 (11.1%)	24 (88.9%)	
Ulcer	No	44 (49.4%)	45 (50.6%)	<0.001^C^*
	Yes	0	41 (100%)	
Bleeding	No	44 (38.6%)	70 (61.4%)	<0.001^C^*
	Yes	0	16 (100%)	
Deep vein thrombosis	No	44 (33.8%)	86 (66.2%)	-

We found that absolute neutrophil counts (CI 95%), absolute lymphocyte counts (CI 99%), and calculated NLRs were statistically significant inflammatory markers related to primary varicose veins in both groups (Table 2).

**Table 2:** Distribution of subjects based on absolute neutrophil count, absolute lymphocyte count, and NLR in groups 1 and 2 ^t^t-test. ^MW^Mann-Whitney U test. *Statistical significance. Both groups had a significant distribution in the means of absolute neutrophil counts and absolute lymphocyte counts on t-test analysis. There is a significant difference in mean distribution of absolute lymphocyte counts between the two groups.

Variable	Groups				p-Value
	Group 1		Group 2		
	Mean ± SD	Median (min, max)	Mean ± SD	Median (min, max)	
Absolute neutrophil count x 10,000	3.9 ± 1.11	3.69 (2.05, 8.16)	4.41 ± 1.38	4.41 (1.53, 6.87)	0.0168^t^*
Absolute lymphocyte count x 10,000	2.28 ± 0.61	2.20 (1.12, 3.94)	1.92 ± 0.62	1.9 (0.72, 3.56)	<0.001^t^*
NLR	1.9 ± 0.7	1.8 (1, 3.9)	2.5 ± 1.2	2.2 (1, 7.8)	<0.001^MW^*

**Figure 1:**
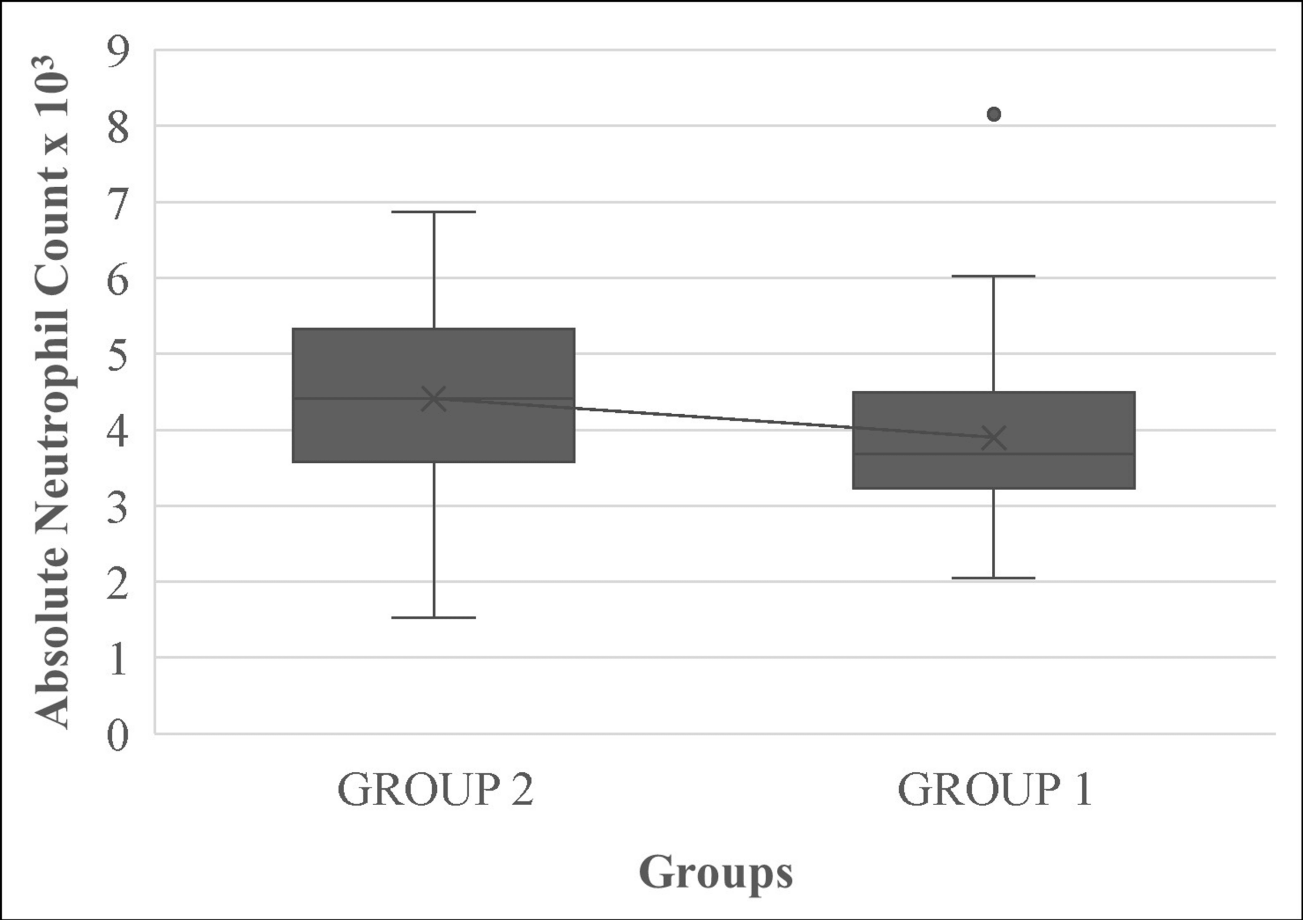
Mean plot of absolute neutrophil counts in groups 1 and 2

**Figure 2:**
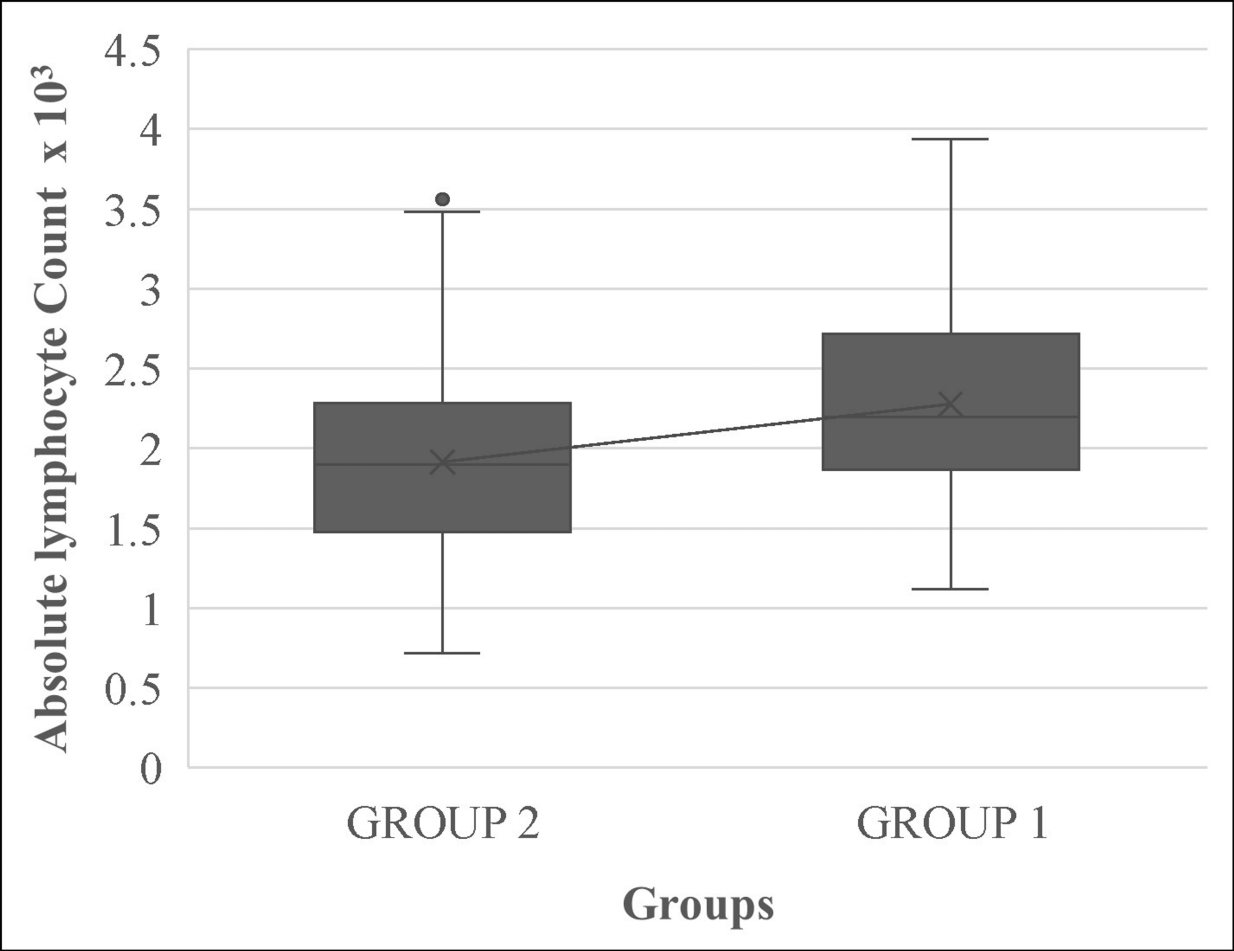
Mean plot of absolute lymphocyte counts in groups 1 and 2

NLRs of both patient groups had a strong statistically significant association with the diagnosis of primary varicose veins (p<0.001). In group 1 patients, with lower-grade primary varicose veins, the mean absolute neutrophil counts were lower than the mean absolute lymphocyte counts. In contrast, group 2 patients displayed higher-grade primary varicose veins, with the mean absolute neutrophil counts higher than the mean absolute lymphocyte counts (Figures 1, 2).

**Figure 3:**
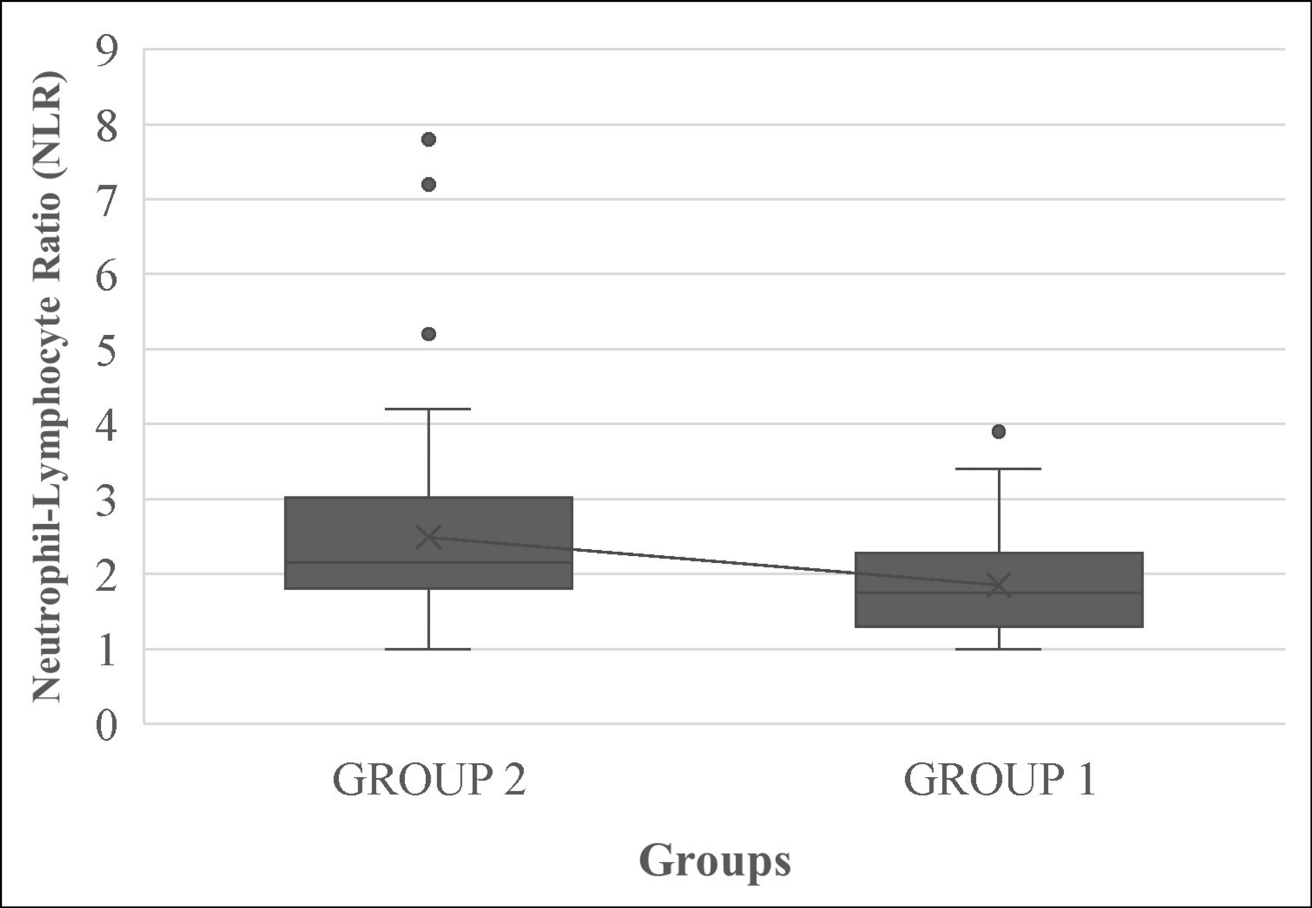
Mean plot of neutrophil-lymphocyte ratio in groups 1 and 2

Therefore, NLRs were lower in group 1 patients than in group 2 patients (Figure 3).

Thus, more diseased veins were associated with higher NLRs.

## Discussion

Varicose veins, clinically described as >3 mm subcutaneous dilated tortuous purplish-blue veins, are caused by incompetent venous valves [4]. Classically, in medicine, varicose veins were long regarded as a common benign medical condition.

In 1994, an internationally recognized classification system called CEAP was created by the American Venous Forum to help accurately group chronic venous disorder (CVD) in patients. CEAP classification incorporates clinical, etiologic, anatomical, and pathophysiologic elements to describe a patient’s type and severity of CVD. The complete range of functional venous abnormalities is described as a CVD. Chronic venous insufficiency (CVI) refers to long-term venous complications such as venous ulceration, venous stasis dermatitis, and edema. The CEAP system is used worldwide for standardized reporting of CVD today [4].

For hundreds of years, these unsightly veins have been treated by “stripping” away these diseased veins. Conventional treatment of varicose veins involves surgical ligation at the saphenofemoral junction (SFJ) and stripping or removal of the superficial vein. Nowadays, the serious operative risks associated with vein stripping favor minimally invasive endovenous techniques such as radiofrequency ablation (RFA). RFA is associated with fewer postoperative complications. Although surgical treatment options have come a long way, the risks of post-operative complications and recurrence remain.

Though we have developed various methods to treat varicose veins over the years, to date we have yet to fully comprehend its cause. We do, however, know that factors, such as obesity, multiparity, use of estrogen therapy, family history of CVD, smoking, and deep vein thrombosis, increase the likelihood of CVD development [2]. Varicosities that are not associated with known predisposing factors are considered “primary varicose veins.” In a large-scale epidemiologic analysis study of 18,560 volunteers, Chiesa et al. used superficial venous system Doppler ultrasound imaging and found that worse visible disease positively correlated with increased frequency of venous valve incompetence. Interestingly, however, sometimes the reported symptoms did not correlate with functionally abnormal veins, and other times asymptomatic individuals were found to have venous incompetence [2].

The pathophysiology behind the development of chronic venous disease is not yet well understood and is under research. The leukocyte “trapping” model is the most widely accepted theory today [1]. Inflammatory mediators modulate a sequence of events wherein altered hemodynamic flow forces lead to venous stasis and hypertension. As a result, leukocytes enter the venous wall and valves activating wall remodeling and valvular destruction [3,5-7].

Contrary to traditional medical beliefs, varicose veins are not simply a common benign condition. Varicose veins have been found to cause not just great morbidity but also an increased risk of mortality. A cohort study conducted by Wu et al. found an associated 1.37 times increased risk of mortality (95% CI 1.19 to 1.57; p<0.0001) and an attributable 2.05 times greater risk of major adverse cardiovascular events such as heart failure, acute coronary syndrome, ischemic stroke, and venous thromboembolism. The study thereby warns that varicose veins must not be overlooked and do require medical attention and treatment [8]. With this forewarning in mind, our study aims to help future physicians hopefully take preemptive action with a simpler more quantitative screening tool such as NLR.

The cause for the development of this venous incompetence in individuals who otherwise have no risk factors was investigated in a 2016 study by Lattimer et al., who took peripheral venous blood samples from the ankle region of CVI patients. These blood samples had markedly increased inflammatory cytokine markers of interleukin (IL)-6, IL-8, and MCP-1. Thus, an inflammatory process is involved in varicose veins development [9].

In 2020, Tiwari et al. found that phlebotomy blood drawn from the site of a varicose vein had a significantly increased concentration of IL-6, fibrinogen, and hemoglobin compared with patients’ antecubital blood sample [10]. Thus, the inflammation described by Lattimer et al. was found to be increased in tissues drained by the varicose vein itself [9,10]. Therefore, researchers concluded that a localized inflammatory pathophysiologic process is associated with the development of venous disorders.

A cohort study of 174 patients with CVI conducted by Engin and Goncu determined that NLR and plateletcrit were independent predictors of clinical severity of superficial CVI [11]. In another study, Tiwari et al. found that the dual inflammatory markers - NLR and IL-6 - could be used together to predict primary varicose veins with high levels of sensitivity and specificity [6].

Our study builds upon the progress thus far in understanding the pathophysiology of CVD. In this study, we aimed to look at NLR alone for its accuracy as an independent predictor of primary varicose veins. Inflammation and higher neutrophil counts were found to be significant in patients with a more severe stage of disease at presentation. Therefore, a higher NLR was accurately prognostic of a higher degree of varicose veins.

In Turkey, Budak followed up at four specific interval times post-endovascular ablation of patients with CEAP grade 2-4 varicose veins and concluded that NLR was a good prognostic indicator of perioperative outcomes. NLR was found to be predictive of complications in these patients with a sensitivity of 75% and specificity of 62%. Higher NLR in these post-operative patients correlated with partial recanalization of the ablated vein, thereby garnering a poorer treatment outcome [12].

The results observed in Budak’s study are within reasonable hypothetical concordance with our study, which shows that patients who suffered from more severe CVD had higher NLRs. Budak’s study proves that it is no unreasonable inference that patients with more severe disease are likely to have a higher rate of disease recurrence post-ablation treatment [12]. Linking both our study findings, researchers can infer that NLR can be used to screen patients for primary varicose vein development. Asymptomatic or post-ablation patients with higher ranges of NLR may be at risk of developing or may already have chronic venous disease.

However, multiple study variables may be improved upon to gain better insights. To propose a test as a screening method, it is necessary to calculate the true and false positives and negative rates. This has not been done in this study, and therefore its suitability as a screening test cannot be determined conclusively. This study has no control group, which is a major deficiency and severely limits the interpretation of the data and the conclusions that can be drawn. Furthermore, as NLR is a non-specific marker of inflammation, there are likely multiple inflammatory conditions unrelated to varicose veins, which could produce abnormal results, confounding the viability of this study. A multivariate analysis would be required to assess other inflammatory and/or other co-existing medical conditions. Moreover, 130 varicose vein patients recruited over seven years represent approximately 18 patients a year, which does not correlate with most global statistics on the approximate prevalence of varicose veins. Patients in the region where this single-center study was conducted do not prioritize varicose veins as a serious condition or are unaware that they are potentially suffering from such a disease. Thus, there are very few patients who seek medical or surgical help concerning the symptomology of varicose veins. True numbers of patients with varicose veins disease are estimated to be far higher than the hospital database accounts for. Expanding patient diversity and the geographic location pool would greatly increase the strength of the findings within this study. A CEAP classification was used to assess severity. Though CEAP is useful, it is a basic assessment tool. For increased strength in assessment, additional methods such as the Venous Clinical Severity Score, the Venous Segmental Disease Score to define the venous anatomy, the Venous Disability Score, The Aberdeen Varicose Vein questionnaire, and the Chronic Venous Insufficiency Questionnaire can be used [13]. A standardized color Duplex ultrasonography assessment protocol could also provide a better assessment of patients included in the study.

Thus, aggregating the limitations of this small “proof of concept” study is based on incomplete retrospective data. Though the concept is intriguing, it requires a much larger prospective study with appropriate protocols and controls to truly be put into action by physicians globally. While being mindful of these factors, our study preemptively finds that NLR could be considered to effectively be used in screening for and tracking the incidence of primary varicose veins in patients after further supportive studies and analyses.

A stepping stone for future researchers, the results of this study may be used to help unravel the mystery surrounding the pathophysiology of primary varicose veins and the role of inflammation in the disease occurrence and progression. This knowledge will help better identify which patients are at risk for developing progressive primary venous disease and which patients will require prevention or specialized treatment options. Future study findings can be used to diagnose and prevent venous disease at much earlier stages than typically done today, reducing the burden of CVD morbidity and mortality risk. Tailoring the best preventative measures or treatment options to each unique primary varicose vein patient’s prognosis may one day be the practical approach to addressing CVD.

## Conclusions

NLR is easily calculated from routine blood tests and therefore is a low-cost parameter to check in patients. A higher NLR is prognostic of a higher degree of varicose veins. Thus, it provides a simple, cost-effective method for screening patients for primary varicose veins. This simple screening can reduce the worldwide burden of undiagnosed and untreated varicose veins, thereby providing relief and reduced morbidity from this common pervasive disease process. In this study, we built upon past studies that used NLR with other parameters in the risk analysis of primary varicose veins. This study finds that there is a possibility that NLR may provide strength to the screening and risk diagnosis for primary varicose veins. Furthermore, NLR may provide valuable insight for research studying the pathophysiology of primary varicose veins.
